# Early Screening of Sleep-Disordered Breathing Using Metaheuristic-Optimized Extreme Learning Machines

**DOI:** 10.3390/diagnostics16132050

**Published:** 2026-06-30

**Authors:** Thaer Thaher, Alaa Sheta, Huthaifa I. Ashqar, Hamouda Chantar, Salim Surani

**Affiliations:** 1 Department of Computer Systems Engineering, Arab American University, Jenin P.O. Box 240, Palestine; 2 Department of Computer Science, Southern Connecticut State University, New Haven, CT 06515, USA; 3 Computer Science Department, Nile University, Cairo 12677, Egypt; 4 Department of AI and Data Science, Arab American University, Jenin P.O. Box 240, Palestine; huthaifa.ashqar@aaup.edu; 5 AI Program, Columbia University, New York, NY 10027, USA; 6 Faculty of Information Technology, Sebha University, Sebha 18758, Libya; ham.chantar@sebhau.edu.ly; 7 Department of Medicine, University of Houston, Houston, TX 77004, USA; srsurani@gmail.com

**Keywords:** obstructive sleep apnea, sleep-disordered breathing, extreme learning machine, metaheuristic optimization, machine learning, screening

## Abstract

**Background/Objectives**: Obstructive sleep apnea (OSA) is a common and serious sleep-related disorder that causes repeated interruptions in breathing during sleep. Traditional diagnostic methods, such as polysomnography, are accurate but costly, time-consuming, and unsuitable for large-scale screening. This study proposes and evaluates a lightweight diagnostic framework based on an Extreme Learning Machine (ELM) optimized by a set of basic and advanced metaheuristic optimizers. The model aims to evaluate whether metaheuristic optimization can improve ELM-based classification performance using structured demographic, clinical, and sleep-related predictors. **Methods**: Two real datasets were employed to train and evaluate the proposed framework: (i) a clinical OSA dataset with 274 subjects and 31 demographic/anthropometric and sleep-related predictors, and (ii) a public strongly imbalanced Sleep-Disordered Breathing (SDB) dataset with 500 subjects and 10 structured predictors. Metaheuristic algorithms are used to optimize ELM weights and biases, addressing the instability of random initialization and improving model generalization. The optimized models are evaluated against eight baseline classifiers, including logistic regression (LR), k-nearest neighbors (KNN), decision tree (DT), random forest (RF), support vector machine (SVM), multilayer perceptron (MLP), XGBoost (XGB), and a standard ELM classifier. **Results**: Results show that metaheuristic optimization moderately improves ELM on the OSA dataset, increasing ROC-AUC from 0.6527 to about 0.73 and accuracy from 0.6573 to about 0.69–0.70, while on the highly imbalanced SDB dataset, it yields modest ROC-AUC gains (from 0.5132 to about 0.544–0.548) with small decreases in accuracy and F1-score. We additionally assess class-imbalance handling on the SDB dataset and analyze feature importance with permutation importance and SHAP, which shows the models rely heavily on diagnosis-derived predictors. **Conclusions**: The proposed framework provides a lightweight ELM-based decision-support approach with low inference cost after offline optimization. The results suggest potential value for screening-oriented OSA/SDB classification, but further validation with larger cohorts and a screening-only feature set is needed before clinical implementation.

## 1. Introduction

Obstructive sleep apnea (OSA) is a common and serious sleep-related breathing disorder marked by repeated partial or complete obstruction of the upper airway during sleep, with symptoms such as loud snoring, daytime sleepiness, and morning headaches [1,2,3]. It affects more than 100 million people worldwide, and untreated OSA is strongly associated with cardiovascular disease, hypertension, diabetes, and a higher risk of workplace and traffic accidents, with an estimated economic burden exceeding 140 billion per year in the United States alone [4,5,6,7]. These figures underline the urgent need for accurate and accessible screening solutions.

Polysomnography remains the diagnostic standard but is expensive, time-consuming, and impractical for large-scale screening, and many automated models rely on physiological signals such as airflow, oxygen saturation, and electrocardiogram-derived features, whose acquisition requires specialized equipment and controlled conditions [8]. Simpler structured predictors such as age, body mass index, snoring, neck circumference, and daytime sleepiness correlate strongly with OSA and have been widely used with machine learning models for low-cost screening and risk stratification [9,10,11].

Extreme Learning Machine (ELM) is a lightweight single-hidden-layer learning model known for fast training and simple implementation [12,13]. However, the standard ELM randomly initializes the input-to-hidden weights and hidden biases, which can lead to unstable performance across runs and sensitivity to the initial parameter configuration [14,15]. Metaheuristic optimization can reduce this sensitivity by searching for better hidden-layer parameters before computing the output weights analytically [16]. However, most existing OSA/SDB studies use a single optimizer, a limited set of baseline models, or only one dataset. Therefore, further empirical comparison is needed to evaluate which optimization strategies are most useful for ELM-based OSA/SDB classification under different data conditions, including class imbalance.

Motivated by these considerations, this study evaluates a metaheuristic-optimized ELM framework for OSA/SDB classification using structured demographic, anthropometric, clinical, polysomnographic, and cardiorespiratory predictors. The contribution is primarily empirical: we compare eleven metaheuristic optimizers integrated with ELM under a common experimental protocol and benchmark them against standard machine learning classifiers on two datasets. This design allows us to assess when metaheuristic optimization improves the standard ELM and when its benefit is limited by data characteristics such as class imbalance. The main contributions are summarized as follows:

1. We evaluate a lightweight ELM-based classification framework using structured demographic, anthropometric, clinical, polysomnographic, and cardiorespiratory predictors from two OSA/SDB datasets.
2. We systematically integrate eleven metaheuristic algorithms covering evolutionary, math-based, physics-based, and swarm-based families to optimize ELM hidden-layer weights and biases under a common objective and protocol.
3. We perform an extensive empirical study on two real datasets, benchmarking all metaheuristic-optimized ELM variants against standard ML baselines and we analyze predictability with permutation importance and SHAP to identify the predictors that drive the models.
4. We examine whether optimized ELM variants improve the standard ELM and how their performance compares with conventional classifiers under different class-balance conditions.

The remainder of this paper is organized as follows: Section 2 reviews related works. Section 3 describes the datasets, preprocessing steps, the proposed optimized-ELM framework, and the experimental setup and evaluation measures. Section 4 presents the experimental results for both baseline classifiers and metaheuristic-optimized ELM models. Section 5 discusses the findings and limitations. Finally, Section 6 summarizes the main findings, outlines practical implications, and suggests directions for future research.

## 2. Related Works

### 2.1. OSA Detection Using Demographic and Clinical Data

Traditional early identification of obstructive sleep apnea conditions depends on clinical evaluations and demographic information, which provide accessibility and practicality to these features. It has been discovered that factors such as neck circumference, age, BMI, and gender, along with other lifestyle indicators including smoking or alcohol consumption, have been highly correlated with the risk of OSA problems [17,18]. Clinically observed symptoms such as habitual snoring, excessive daytime sleepiness, and apneas during sleep are also common indicators used in screening processes for diagnosing OSA. In contrast to the monitoring of physiological signals, easily collected clinical and demographic information provides a low-cost, non-invasive alternative for stratifying the risk of OSA [19,20,21]. These variables offer advantages over large-scale screening and resource-constrained contexts, where access to sleep laboratories or specialized tools is limited. Although clinical and demographic variables are useful for risk stratification, their discriminative ability is often limited when they are used without additional modeling or physiological information [22,23]. This drawback has motivated the authors of this work to integrate machine learning models to enhance the accuracy of early prediction of OSA by identifying complex, nonlinear interactions and dependencies among OSA features. This finding aligns with recent work in interpretable machine learning, where researchers have used clinical features and questionnaire data to predict severe OSA risk and, at the same time, pinpoint the risk factors that matter most [24].

### 2.2. Machine Learning and Deep Learning Models in OSA

The widespread availability of medical data has encouraged researchers to adopt deep learning and machine learning techniques for OSA detection [25,26,27,28]. Conventional machine learning algorithms, such as support vector machines, logistic regression, and decision trees, as well as ensemble learning models, have been used to classify OSA severity based on physiological, demographic, and clinical features [10,29,30,31]. These algorithms can efficiently handle multidimensional data, learn feature interactions, and deliver accurate classification results. Also, the adoption of deep learning and neural networks has been increasing rapidly, providing automatic extraction of valuable hierarchical patterns from structured data and basic physiological signals [20,32,33]. Traditional and recurrent architectures, such as Recurrent Neural Networks (RNNs), are promising for analyzing airflow, electrocardiogram, and oxygen saturation signals. Despite their robust predictive power, deep learning models often require substantial computational resources, large amounts of training data, and careful hyperparameter tuning. However, deep learning models may be difficult to interpret, and the physiological signals required by many of these models can be costly or inconvenient to acquire in routine screening settings. In this regard, lightweight learning models such as the ELM have been adopted as alternatives to deep learning models due to their effective balance between prediction accuracy and computational cost, making them appropriate for highly scalable screening applications [34,35,36].

### 2.3. Metaheuristic-Based Optimization of ELM

Extreme Learning Machines are preferred due to their fast training speed and robust generalization power. However, their performance heavily relies on the initial configuration of hidden-layer weights and biases [34,37,38]. Random initialization of weights and biases may lead to unsatisfactory solutions, unsteady convergence, and contradictory accuracy across runs. To overcome this problem, metaheuristic optimization techniques can be employed to guide the initialization and training of ELMs. Metaheuristics, such as swarm intelligence techniques, are designed to efficiently explore the search space, avoid premature convergence, and find near-optimal solutions for complex optimization problems. Incorporating optimization techniques with ELM models improves learning stability, avoids overfitting, and boosts classification accuracy [34,37,38]. For OSA/SDB classification, metaheuristic-optimized ELM models are worth evaluating because they combine the computational simplicity of ELM with a search process that can reduce sensitivity to random hidden-layer initialization. However, their practical value depends on whether the improvement in test performance justifies the additional offline training cost and whether the gains remain stable across datasets.

Taken together, prior work suggests that OSA/SDB prediction can benefit from combining accessible patient variables with machine learning models that capture nonlinear feature interactions. Within this context, the present study evaluates whether metaheuristic optimization can improve ELM performance under a common experimental protocol.

## 3. Materials and Methods

### 3.1. Dataset Description

This study relies on two real clinical datasets to train and evaluate the proposed models for early detection of sleep apnea and sleep-disordered breathing. Both datasets include a binary diagnosis label and a set of demographic, anthropometric, and physiological features, but they differ in sample size, number of predictors, and class balance.

The first dataset is a clinical dataset of OSA derived from a full-night clinical study [39]. The processed version used here consists of 274 patients and 31 input variables, together with a binary outcome indicating the presence of OSA (class = 1) or its absence (class = 0). The dataset contains 125 positive and 149 negative cases, so it is only moderately imbalanced. The features cover demographic and anthropometric information, comorbidities, questionnaire-based indicators, and polysomnographic indices.

The second dataset is the Sleep-Disordered Breathing (SDB) detection dataset, which contains 500 subjects collected from a publicly available Kaggle resource [40]. From the 18 original variables (including free-text fields), we retain 10 structured predictors and a binary outcome; the target Diagnosis_of_SDB is recoded so that mild, moderate, and severe cases are positive and the remaining subjects are negative. This yields 381 positive and 119 negative samples, a strong class imbalance of about 76% positive cases.

Table 1 presents a summary of the two datasets used in this study, while Table 2 provides a unified overview of all 38 unique predictor variables across the two datasets, indicating for each its type, the dataset(s) in which it appears, and a brief description. In both datasets, all predictors are treated as numeric inputs to the models; the details of scaling, encoding, and any sampling strategies are described in Section 3.2.

### 3.2. Data Processing

For both datasets, we used a simple, consistent preprocessing pipeline. We separated predictors *X* and labels *y*, cleaned the data, removed non-informative fields, and standardized all input features using z-score scaling. All steps were applied identically for baseline models and the metaheuristic-optimized ELM.

#### 3.2.1. OSA Dataset Preprocessing

For the OSA dataset, we used the processed version provided by the original authors [39], which already contains numeric predictors and a binary label (class = 1 for OSA, 0 for non-OSA). No missing values were present, and all 274 records and 31 input features were kept for analysis. Before model training, each feature was standardized on the training split using the z-score transform.

(1) $${\tilde{x}}_{ij}=\frac{x_{ij}-\mu_{j}}{\sigma_{j}},$$

where $\mu_{j}$ and $\sigma_{j}$ are the mean and standard deviation of the feature *j* computed from the training data only, to avoid data leakage and preserve a strict train–test separation (i.e., preventing the preprocessing step from using any information from the evaluation split). The same transformation was then applied to the corresponding test data using these fixed parameters, which also reflects the real-world setting where future/unseen data are unavailable when estimating scaling statistics. Binary and ordinal variables were kept in their numeric form and scaled in the same way.

#### 3.2.2. SDB Dataset Preprocessing

For the SDB dataset, we started from the publicly available Kaggle file [40]. The original data contain 500 subjects, 18 columns, and no missing values. We removed identifier, treatment, and free-text columns and retained ten structured predictors: Age, Gender, BMI, Snoring, Oxygen_Saturation, AHI, ECG_Heart_Rate, ${\mathrm{SpO}}_{2}$, Nasal_Airflow, and Chest_Movement.

The original Diagnosis_of_SDB field includes four categories (Mild, Moderate, Severe, and no SDB). We converted it into a binary label, mapping Mild/Moderate/Severe to the positive class and the remaining subjects to the negative class. In the final processed file, Diagnosis_of_SDB = 1 indicates SDB and 0 indicates non-SDB, giving 381 positive and 119 negative samples.

We then standardized all ten predictors using the same z-score formula in Equation (1), with $\mu_{j}$ and $\sigma_{j}$ estimated on the training data and reused for the test data.

### 3.3. Proposed Optimized-ELM Framework

**Figure 1:**
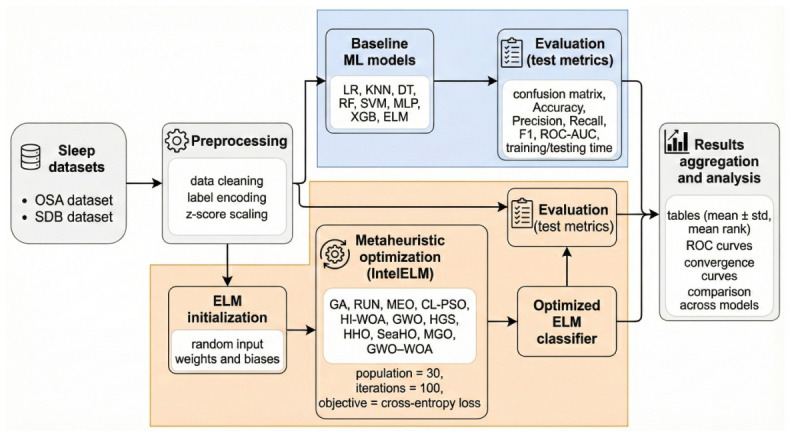
Schematic view of the pipeline and system flow.

The proposed pipeline begins with the two datasets (OSA and SDB), followed by data cleaning and preprocessing (label encoding, and z-score scaling). The processed data are then used in two branches. In the first branch, baseline classifiers are trained and evaluated to provide a reference performance level. In the second branch, the same preprocessed data are passed to the IntelELM framework, where different metaheuristics search for improved ELM weights and biases. In both branches, models are trained and tested under repeated experiments, and the resulting metrics are collected for analysis. Figure 1 illustrates the overall workflow from data collection to model evaluation and result analysis.

#### 3.3.1. Standard ELM Classifier and Mathematical Formulation

The standard ELM is a single-hidden-layer feedforward network whose input weights and hidden biases are assigned randomly, with the output weights computed analytically through the Moore–Penrose generalized inverse rather than by iterative gradient descent [15,41,42]. This yields fast training and good generalization while avoiding the local-minima and slow-convergence problems of backpropagation [16].

**Figure 2:**
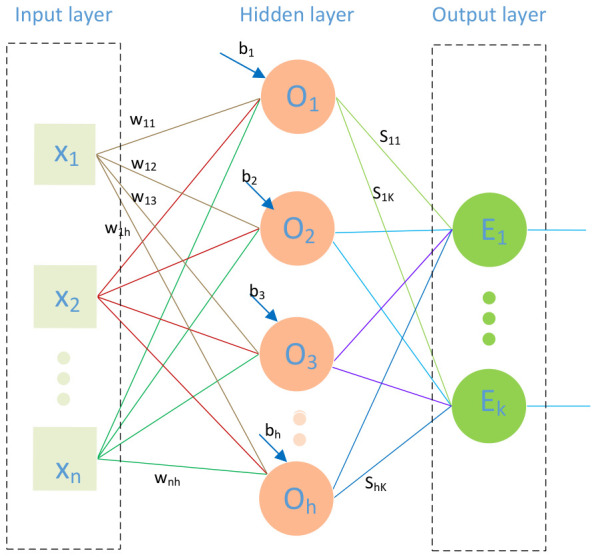
Basic architecture of a single-hidden-layer ELM model.

ELM initially assigns random weights and biases to the input layer and subsequently determines the output-layer weights based on these random values. Typically, the SLNN model has *n* input-layer neurons, *h* hidden-layer neurons, and *k* output-layer neurons. The architecture of this model is illustrated in Figure 2. As presented in [15], the activation function of SLNN can be written as in Equation (2).

(2) $$E_{i}=\sum_{j=1}^{h}S_{j}f\left(w_{j},b_{j},X_{j}\right)$$

where $w_{j}$ indicates the input weight and $b_{j}$ denotes the bias of jth the hidden neuron. The inputs are represented by $X_{j}$, while *E* indicates the output of the SLNN model. Equation (3) represents the matrix of Equation (2).

(3) $$E^{T}=OS$$

where $S={\left[S_{1},S_{2},\ldots ,S_{h}\right]}^{T}$. $E^{T}$ denotes the transpose of matrix *E*. *O* denotes the output matrix of the hidden layer. It is calculated as follows:

(4) $$O={\left[\begin{array}{cccc} f(w_{1},b_{1},X_{1}) & f(w_{2},b_{2},X_{1}) & \ldots & f(w_{h},b_{h},X_{1})\\ \ldots & \ldots & \ldots & \ldots \\ f(w_{1},b_{1},X_{\beta }) & f(w_{2},b_{2},X_{\beta }) & \ldots & f(w_{h},b_{h},X_{\beta }) \end{array}\right]}_{\beta \times h}$$

The principal objective of training is to reduce the error or variance of the ELM. The activation function must be infinitely differentiable in the standard ELM; nonetheless, ELM training results in the determination of the output weight (*S*) by improving the least-squares function as specified in Equation (5). The corresponding output weights are calculated analytically using the Moore–Penrose generalized inverse, as implemented in ELM (see Equation (6)), rather than through iterative adjustment.

(5) $$\underset{S}{min}||OS-E^{T}\left|\right|$$

(6) $$\hat{E}=O^{∔}E^{T}$$

where $O^{∔}$ denotes the generalized Moore–Penrose inverse of the *O* matrix.

#### 3.3.2. Optimization Methodology

Metaheuristic optimization techniques can be used to optimize the weights and biases of the hidden layer in ELM, rather than relying on random assignment of their values. The purpose of optimization is to improve ELM effectiveness and reduce sensitivity to randomly initialized hidden-layer parameters. A metaheuristic optimizer (e.g., PSO, GA) can be used to find the best or near-optimal values for the ELM’s input-to-hidden layer weights and hidden layer biases. After the hidden-layer parameters are optimized, the ELM follows its standard training procedure by computing the hidden-layer outputs and estimating the hidden-to-output weights using the Moore–Penrose inverse.

To optimize the weights and biases of the hidden layer in ELM using a metaheuristic algorithm, two main design aspects must be considered: firstly, the design of the search agent, which solves the ELM’s parameter fine-tuning problem; then, the choice of an appropriate objective function for assessing the quality of the generated solutions (weights and biases).

##### Solution Encoding

**Figure 3:**
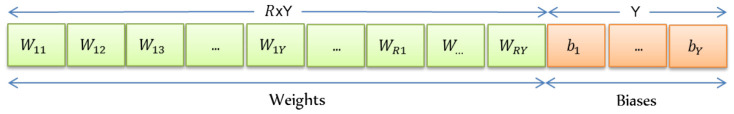
Search agent structure.

To optimize the ELM, we encode all input weights and hidden biases into a single continuous vector $z\in R^{D}$, where *D* is the total number of trainable ELM parameters. For a given candidate *z*, the hidden-layer matrix H(z) is constructed, the output weights β(z) are computed in closed form, and the resulting model is evaluated on the training data. In all experiments, each component of *z* is constrained to lie in the interval [−1, 1], which defines the lower and upper bounds of the search space for the metaheuristics. Figure 3 depicts the encoding schema of the search agent. The search agent has *D* components based on the number of input weights and the number of hidden biases, as shown in Equation (7):

(7) D=R×Y+Y

where *R* denotes the number of weights, and *Y* refers to the number of hidden biases.

##### Objective Function

The fitness (objective) function used by all metaheuristics is the average binary cross-entropy loss on the training set:

(8) $$L\left(z\right)=-\frac{1}{N}\sum_{i=1}^{N}\left[y_{i}\log p_{i}\left(z\right)+\left(1-y_{i}\right)\log\left(1-p_{i}\left(z\right)\right)\right],$$

where *N* is the number of training samples, $y_{i}\in \left\{0,1\right\}$ is the true label of sample *i*, and $p_{i}\left(z\right)$ is the predicted probability of the positive class produced by the ELM with parameters *z*.

The goal of each metaheuristic is to find the parameter vector that minimizes the training loss:

(9) $$z*=\arg\min_{z\in R^{D}}L\left(z\right).$$

This common objective allows a fair comparison of different metaheuristics under the same training criterion. Binary cross-entropy was selected because it provides a continuous probabilistic loss that can be minimized by population-based optimizers. However, this objective does not directly optimize clinical screening metrics such as ROC-AUC, F1-score, sensitivity, or specificity. Therefore, the optimized models were not selected based on training loss alone; their final performance was interpreted using test-set metrics, as described in Section 3.4.

#### 3.3.3. Integration of Metaheuristics with ELM

**Figure 4:**
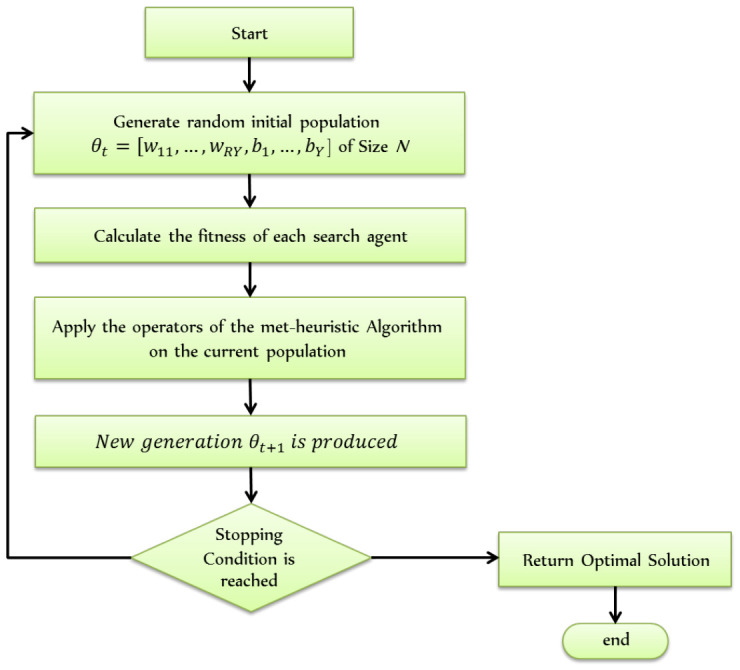
Integrating ELM model with metaheuristic algorithm.

The integration of metaheuristics with ELM follows a unified procedure. Each algorithm starts by generating an initial population of candidate vectors *z*. In each iteration, the current population is decoded into ELM parameters, the cross-entropy loss L(z) is computed for each candidate, and the metaheuristic’s update rules are applied to produce a new population. This process is repeated for a fixed number of iterations (100), with population size 30 in all experiments. At the end of the run, the candidate with the best fitness is selected, and the corresponding ELM (with its optimized weights and biases) is used as the final classifier for that run. The inference phase remains as efficient as a standard ELM, since only a single forward pass through the optimized network is required for prediction. Figure 4 depicts the general flowchart of applying any metaheuristic algorithm for optimizing the parameters of the ELM model.

### 3.4. Experimental Setup and Evaluation Protocol

This subsection describes how the datasets were split, how the baseline and metaheuristic-based models were configured, and how the evaluation procedure was carried out.

#### 3.4.1. Data Splitting and Repeated Experiments

For each dataset, we used a stratified train–test split to preserve the original class proportions, with 80% of the samples for training and 20% for testing in each run. All experiments were repeated 20 times with different random seeds, and the same splits were reused across all models within a run to ensure fair comparison. Feature scaling (z-score standardization) was applied only to the training set and then to the corresponding test set.

#### 3.4.2. Baseline Classifiers and Hyperparameters

We evaluated eight baseline models: logistic regression (LR), k-nearest neighbors (KNN), decision tree (DT), random forest (RF), support vector machine (SVM) with Radial Basis Function (RBF), multilayer perceptron (MLP), XGBoost (XGB), and a standard ELM classifier. All models were implemented using scikit-learn (version 1.6.1) and XGBoost (version 2.1.4) libraries, except ELM, which was implemented via the Intelligent Metaheuristic-based ELM (IntelELM) framework (version 1.3.0) [43,44]. The baseline models were not optimized through a hyperparameter search. Instead, their hyperparameters were fixed using library defaults and, where specified in Table 3, values commonly used in previous machine learning studies. The same configuration was applied across all repeated runs and both datasets. Since no hyperparameter search was conducted, no validation split or search space was required, and the test set was not used for model selection. This avoids hyperparameter-selection leakage. Probability estimates were enabled when required for ROC-AUC calculation. The main hyperparameters are reported in Table 3; all remaining settings follow the defaults of the corresponding libraries. Therefore, these models should be interpreted as untuned, out-of-the-box baselines rather than fully optimized competitors. This limitation is acknowledged in the study.

#### 3.4.3. Metaheuristic-Optimized ELM Configuration

For the optimized models, we used IntelELM to configure a single-hidden-layer ELM with fixed architecture (number of hidden neurons and activation function) across datasets. Eleven metaheuristic algorithms were employed to optimize the ELM weights and biases. These algorithms include a diverse set of basic and advanced metaheuristics drawn from different families: an evolutionary-based method, Genetic Algorithm (GA) [45]; a math-based optimizer, RUNge Kutta optimizer (RUN) [46]; a physics-based method, Modified Equilibrium Optimizer (MEO) [47]; and a rich group of swarm-based algorithms, including Comprehensive Learning Particle Swarm Optimization (CL-PSO) [48], Hybrid Improved Whale Optimization Algorithm (HI-WOA) [49], standard Grey Wolf Optimizer (GWO) [50], Hunger Games Search (HGS) [51], Harris Hawks Optimization (HHO) [52], Sea-Horse Optimization (SeaHO) [53], Mountain Gazelle Optimizer (MGO) [54], and the hybrid Grey Wolf–Whale Optimization Algorithm (GWO-WOA) [55]. The selection of these particular algorithms was guided by three criteria. The selected optimizers were chosen to cover different metaheuristic families rather than relying on one search strategy. The set includes well-established algorithms, such as GA, PSO, GWO, WOA, and HHO, together with newer methods, including RUN, MEO, SeaHO, and MGO, as well as one hybrid variant. This choice allows the study to compare classical, recent, and hybrid search mechanisms under the same experimental protocol. In addition, since the no-free-lunch principle suggests that no optimizer performs best across all problems, and since many previous ELM-based OSA/SDB studies use only one optimizer, a broader comparison is needed to examine whether the optimizer choice has a meaningful effect on ELM performance.

Each optimizer was run for 100 iterations with a population size of 30, and the cross-entropy loss on the training data was used as the objective function. Algorithm-specific parameters were set to either IntelELM defaults [43] or values suggested in the original papers and were kept constant across all runs. The same optimizer settings were used for all repeated runs, and the test sets were used only for final evaluation after model training.

#### 3.4.4. Class-Imbalance Handling

The SDB dataset is strongly imbalanced, with 76.2% positive cases, whereas the OSA dataset is close to balanced. To examine whether standard techniques change how the models behave under this imbalance, we evaluated three handling strategies on the SDB dataset and compared each with the no-handling baseline used in the main experiments. Every strategy was applied within the same 20 stratified train/test splits, and any modification of the data was limited to the training partition so that the test set remained untouched.

The first two strategies are informed oversamplers applied to the training data. SMOTE creates synthetic minority cases by interpolation and balances the two classes exactly. ADASYN creates them adaptively, in proportion to local difficulty, and therefore yields an approximately balanced training set. The third strategy is decision-threshold optimization. Rather than the default 0.5 cut-off, we selected the threshold that maximizes Youden’s J (sensitivity plus specificity minus one) on an internal stratified 75/25 split of the training data, and then evaluated the model after refitting it on the full training set. The amount of training data is the same as in the no-handling condition, so only the decision rule changes, and the test set plays no part in choosing the threshold.

#### 3.4.5. Evaluation Measures

We evaluated the models using standard classification quality metrics and computational time. All metrics were computed on the training and test sets and then averaged across repeated runs.

##### Confusion Matrix for Binary Diagnosis

For both datasets, the problem is binary:

- Positive class: Patient with obstructive sleep apnea or sleep-disordered breathing (OSA/SDB present).
- Negative class: Patient without OSA/SDB (no sleep apnea).

**Figure 5:**
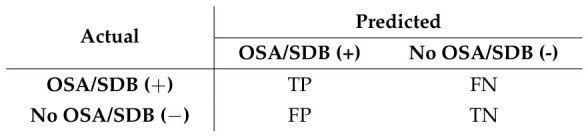
Schematic confusion matrix for the binary diagnosis task. The symbols + and − denote the positive class (OSA/SDB present) and negative class (no OSA/SDB), respectively. TP, TN, FP, and FN denote true positives, true negatives, false positives, and false negatives, respectively.

For a given test set, predictions can be summarized in a confusion matrix as shown in Figure 5, where

- True positive (TP) encompasses correctly predicted OSA/SDB cases;
- True negative (TN) represents correctly predicted non-OSA/non-SDB cases;
- False positive (FP) encompasses predicted OSA/SDB, but the patient is actually non-OSA/non-SDB;
- False negative (FN) represents predicted non-OSA/non-SDB, but the patient actually has OSA/SDB.

##### Classification Quality Metrics

From the confusion matrix, we compute the following measures.

(10) $$\mathrm{Accuracy}=\frac{\mathrm{TP}+\mathrm{TN}}{\mathrm{TP}+\mathrm{TN}+\mathrm{FP}+\mathrm{FN}}.$$

(11) $$\mathrm{Precision}\left(\mathrm{PPV}\right)=\frac{\mathrm{TP}}{\mathrm{TP}+\mathrm{FP}}.$$

(12) $$\mathrm{Recall}\left(\mathrm{sensitivity}\right)=\frac{\mathrm{TP}}{\mathrm{TP}+\mathrm{FN}}.$$

(13) $$F1=\frac{2\times \mathrm{Precision}\times \mathrm{Recall}}{\mathrm{Precision}+\mathrm{Recall}}.$$

Sensitivity and precision are reported under their clinical names: sensitivity (recall) and Positive Predictive Value (PPV), respectively. In addition to the measures used in the main experiments (including ROC-AUC), the imbalance analysis reports two further metrics that are better suited to imbalanced data: the area under the precision–recall curve (PR-AUC) and the Matthews correlation coefficient (MCC). ROC-AUC summarizes the trade-off between true positive rate and false positive rate across all possible decision thresholds. Higher AUC values indicate a better ability to distinguish between OSA/SDB and non-OSA/non-SDB patients.

The PR-AUC, computed via average precision, serves as a vital complement to ROC-AUC when evaluating imbalanced datasets. For a non-informative classifier, the baseline PR-AUC value is mathematically equivalent to the positive-class prevalence (0.762 for SDB). This baseline establishes the critical reference threshold against which the model’s actual performance must be evaluated. For ROC-AUC and PR-AUC, which require a continuous positive-class score, the scikit-learn classifiers use their predicted probabilities, while the ELM and metaheuristic-optimized ELM models, which produce raw least-squares outputs, are converted to a positive-class probability with a row-wise softmax. Because the softmax is monotonic, it does not change the ROC-AUC or PR-AUC values.

The MCC summarizes all four cells of the confusion matrix in a single value within the interval [−1, 1], where zero corresponds to chance:

(14) $$\mathrm{MCC}=\frac{\mathrm{TP}\cdot \mathrm{TN}-\mathrm{FP}\cdot \mathrm{FN}}{\sqrt{\left(\mathrm{TP}+\mathrm{FP})(\mathrm{TP}+\mathrm{FN})(\mathrm{TN}+\mathrm{FP})(\mathrm{TN}+\mathrm{FN}\right)}}$$

##### Computational Time

To assess efficiency, we measure training time and testing time for each model. Training time is the time required to fit the model on the training data including metaheuristic optimization steps when used. In contrast, testing time is the time needed to generate predictions for the test set using the trained model. Both times are recorded for every run and then summarized by their mean and standard deviation. This allows us to compare not only predictive performance but also computational cost across baseline and metaheuristic-optimized ELM models.

For all tables in Section 4 in which boldface is used, bold values indicate the best average result within the corresponding column. Higher values are better for classification metrics, whereas lower values are better for computational time and mean rank.

#### 3.4.6. Environment and Tools

All experiments were implemented in Python 3.9.12 within an Anaconda environment, using a framework developed based on IntelELM and other standard scientific libraries. The experiments were conducted on a Windows 64-bit system with an 11th Gen Intel^®^ Core™ i7-1165G7 CPU @ 2.80 GHz and 16 GB of RAM. All runs were performed on the same machine and under the same software environment to ensure consistent timing measurements and reproducible results.

## 4. Experimental Results

### 4.1. Results and Analysis of Baseline Models

#### 4.1.1. Baseline Results on the OSA Dataset

##### Test Performance

**Figure 6:**
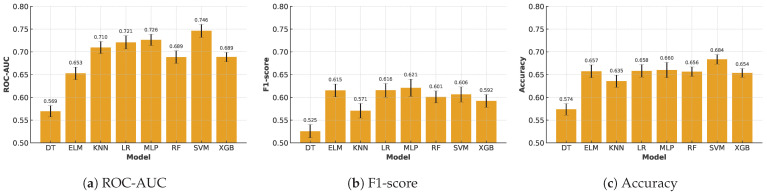
Test performance of the eight classifiers. Bars represent the mean test value over 20 runs, and the vertical lines on each bar show ±1 standard error (SE). The SE is computed as $\mathrm{SE}=s/\sqrt{n}$, where *s* is the std of the metric over the 20 runs (n=20).

As shown in Table 4 and Figure 6, there are noticeable differences in the effectiveness of the compared models. The DT is the weakest baseline, with an ROC-AUC of 0.5693, an F1 of 0.5255, and an accuracy of 0.5736. On the other hand, among the stronger models, SVM and MLP stand out. SVM achieves the highest ROC-AUC (0.7462) and accuracy (0.6836), while MLP obtains the best F1-score (0.6210) and recall (0.6160). Compared with DT as a baseline, SVM improves ROC-AUC by about 31% and accuracy by about 19%, while MLP improves ROC-AUC by about 28% and accuracy by about 15%. The F1-score gains are also notable: around 18% for MLP and 15% for SVM relative to DT. These gains indicate a clear benefit of more advanced models for OSA risk prediction.

Considering the other models, LR, ELM, RF, and XGB form a middle group. LR has balanced performance with accuracy 0.6582, F1-score 0.6155, and ROC-AUC 0.7209, which is close to MLP and SVM but slightly lower. ELM and RF have similar accuracies (around 0.656–0.657) but somewhat lower AUC values (0.6527 and 0.6885). KNN is weaker than these models, mainly due to lower recall (0.5340). The mean rank values, which denote the average ranking of each model across all evaluation metrics, confirm these findings: MLP has the best overall rank (2.0), followed by SVM (2.6), LR (3.2), ELM and RF (4.4), XGB (5.0), KNN (6.4), while DT has the worst rank (8.0).

To demonstrate the variability across the 20 runs and how much the performance fluctuates from run to run, the standard error (SE) lines are visualized in Figure 6. Consistently, SEs are relatively small for all models, which means the results are stable under repeated random splits. However, variability is slightly larger for MLP.

##### Training Performance and Overfitting

**Figure 7:**
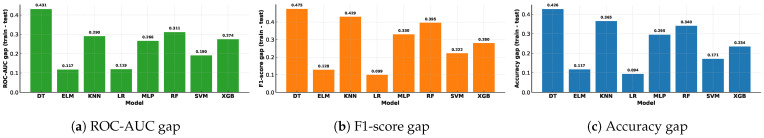
Generalization gaps (train − test) for baseline classifiers on the OSA dataset.

To discuss overfitting and generalization, quantifying gaps between training and test performance for each metric are reported in Table A1 and visualized in Figure 7. From Table A1, we observe that several models fit the training data almost perfectly. DT, KNN, and RF reach training accuracies near 1.0 and ROC-AUC close to 1.0 with zero or very low standard deviation. MLP and XGB also report very high training scores (e.g., MLP training AUC 0.9923, XGB 0.9627). In contrast, ELM, LR, and, to a lesser extent, SVM keep more moderate training scores, which indicates better regularization or less capacity to memorize the data.

For further clarification, Table 5 and Figure 7 summarize these differences through the generalization gap Δ = train−test. DT, KNN, RF, and MLP show large gaps in all metrics. For example, DT has an AUC gap of 0.4307 and an accuracy gap of 0.4264, meaning that more than 40% points of its training performance are lost on the test set. KNN and RF show similar patterns, with AUC gaps around 0.29–0.31 and F1 gaps around 0.39–0.43. MLP also suffers from notable overfitting, with F1 and AUC gaps of around 0.33 and 0.27. These models are powerful but tend to memorize the training set, especially in a relatively small and somewhat imbalanced sample.

In contrast, LR and ELM have the fewest gaps. LR shows an accuracy gap of 0.0939 and an AUC gap of 0.1193; ELM has a similar AUC gap (0.1169) and a slightly larger accuracy gap (0.1167). SVM lies between these two groups, with moderate gaps (AUC gap: 0.1902; accuracy gap: 0.1707). Figure 7 makes this pattern clear: LR and ELM have the shortest bars, SVM sits in the middle, while DT, KNN, RF, MLP, and XGB have much taller bars. This means that LR and ELM offer the best balance between test accuracy and robustness, while SVM trades a bit more overfitting for the highest test-set AUC.

From an OSA screening perspective, this trade-off is essential. SVMs and MLPs achieve the best raw test performance and can be attractive when the focus is on maximizing discrimination. However, their significant gaps suggest that their decisions are more sensitive to the specific training sample. LR and ELM provide slightly lower test metrics but more stable generalization, which may be safer when the model is deployed in new clinical settings.

##### Training and Testing Time

In terms of efficiency, Table 6 reports the average training time (time needed to fit the model on the training data) and testing time (time needed to generate predictions for the test set) over the 20 runs, together with their standard deviations and an overall mean rank (lower is better). We can observe that ELM is the fastest model, with the lowest training time (0.0046 s) and testing time (0.0001 s). DT and LR are also swift, with training and testing times of a few milliseconds. In contrast, MLP, KNN, RF, and SVM are noticeably slower, especially during training for MLP (0.3751 s on average) and during testing for KNN, RF, and SVM, which take longer to produce predictions. Overall, these results indicate that ELM offers a favorable trade-off between predictive performance and computational cost, which is essential for real-time or resource-constrained screening scenarios.

#### 4.1.2. Baseline Results on the SDB Dataset

For the SDB dataset, Table 7 shows that most models achieve relatively high accuracy (0.72–0.76), very high recall (often above 0.9), and strong F1-score, but their ROC-AUC values remain low, mainly in the 0.49–0.58 range. This pattern reflects the strong class imbalance (only 119 of 500 samples are “no sleep apnea”): the classifiers tend to label most cases as having sleep apnea, boosting recall and F1 but offering poor discrimination between the positive and negative classes. XGB and MLP provide the best overall trade-off according to the mean rank, with XGB achieving the highest AUC (0.5804) and tied-best accuracy and F1 with SVM (0.7600 and 0.8636).

Training results in Table A2 confirm that several models almost perfectly fit the training data. When these training results are contrasted with the much lower test AUC values, it becomes clear that many models heavily overfit to the imbalanced SDB dataset: they learn the majority class very well but fail to generalize to the minority “no sleep apnea” cases. Overall, the SDB results highlight that, under severe imbalance, high recall and F1-score alone can be misleading, and ROC-AUC, together with training–testing differences, should be used to assess how reliable the models really are.

Overall, the results on the SDB dataset are broadly consistent with the findings on the OSA dataset, but the impact of class imbalance is much more substantial. In both datasets, simple models such as DT perform worst, while more advanced models (LR, MLP, SVM, XGB) achieve the highest test accuracy and F1-score, confirming that the relative ranking of the classifiers is stable across data sources. However, compared with OSA, all models achieve lower ROC-AUC on SDB, despite higher recall and F1, indicating that the strong skew towards the apnea class makes it harder to distinguish the minority “no apnea” cases. The training results also follow the same pattern: DT, KNN, and RF almost memorize the training set; MLP and XGB achieve very high training scores, while ELM and LR show more moderate training performance, again suggesting better generalization.

### 4.2. Results of Metaheuristic-Optimized ELM

#### 4.2.1. Results on OSA Dataset

**Figure 8:**
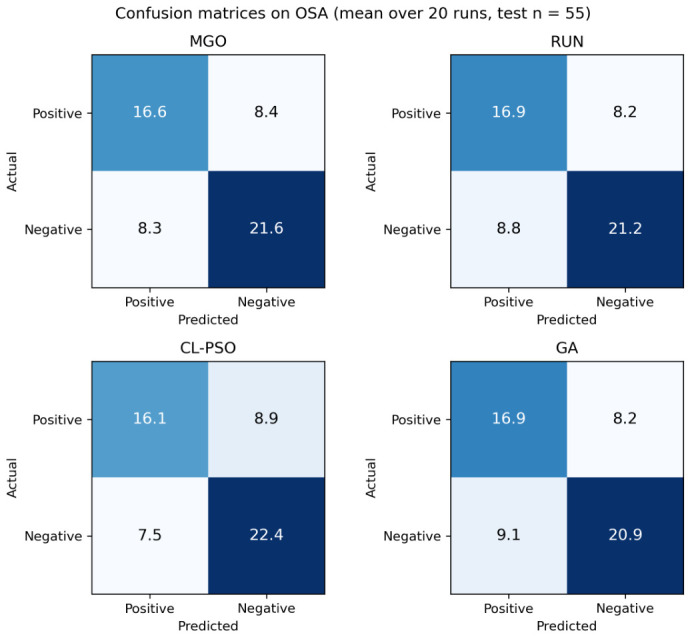
Confusion matrices of the four best optimized ELM models (MGO, RUN, CL-PSO, and GA) on the OSA dataset, averaged over the 20 runs (test n = 55).

**Figure 9:**
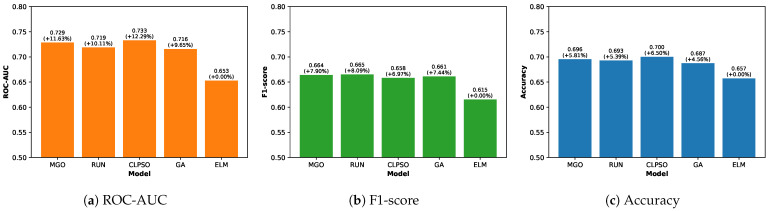
Comparison of the four best-performing metaheuristic-based models (MGO, RUN, CLPSO, GA) against ELM on the OSA dataset. Bars show the actual metric values, and the labels above each bar indicate the corresponding percentage improvement (or decline) relative to ELM.

Figure 8 shows the confusion matrices of the four best optimized ELM models on the OSA dataset, averaged over the 20 runs. Table 8 reports the test-set classification performance of metaheuristic-optimized ELM models on the OSA dataset. The last row reports the baseline (non-optimized) ELM. It is clear from Table 8 and Figure 9 that optimizing ELM with metaheuristics consistently improves test performance compared with the standard ELM. For example, CL-PSO, MGO, RUN, and GA all raise test accuracy from 0.6573 for plain ELM to about 0.69–0.70 (roughly 5–7% improvement) and increase F1 from 0.6153 to about 0.66 (around 7–8% gain). Their gains in ROC-AUC are more pronounced: CL-PSO and MGO reach 0.7329 and 0.7286, respectively, compared with 0.6527 for ELM, corresponding to an improvement of about 11–12%. Even the weaker optimizers, such as HI-WOA and HHO, still provide higher AUC than the non-optimized ELM, although their accuracy and F1 remain closer to the baseline. The mean rank values confirm these trends: MGO, RUN, and CL-PSO achieve the best overall ranks (2.2, 2.6, and 3.0), while the plain ELM and HI-WOA appear among the least competitive methods.

These results indicate that metaheuristic optimization improved the standard ELM on the OSA dataset. The improvement was most visible when the optimized models were compared with the non-optimized ELM, particularly in ROC-AUC, accuracy, recall, and F1-score. However, the optimized ELM variants should not be interpreted as uniformly superior to all baseline classifiers. SVM achieved the highest ROC-AUC among the evaluated models which indicates stronger ranking-based discrimination. In contrast, several optimized ELM variants achieved higher recall and F1-score than SVM, and CL-PSO also achieved higher accuracy. Therefore, the OSA results are better interpreted as a trade-off: SVM provided the strongest ROC-AUC, whereas optimized ELM variants offered competitive threshold-dependent performance while retaining a lightweight inference structure after offline optimization.

#### 4.2.2. Results on SDB Dataset

**Figure 10:**
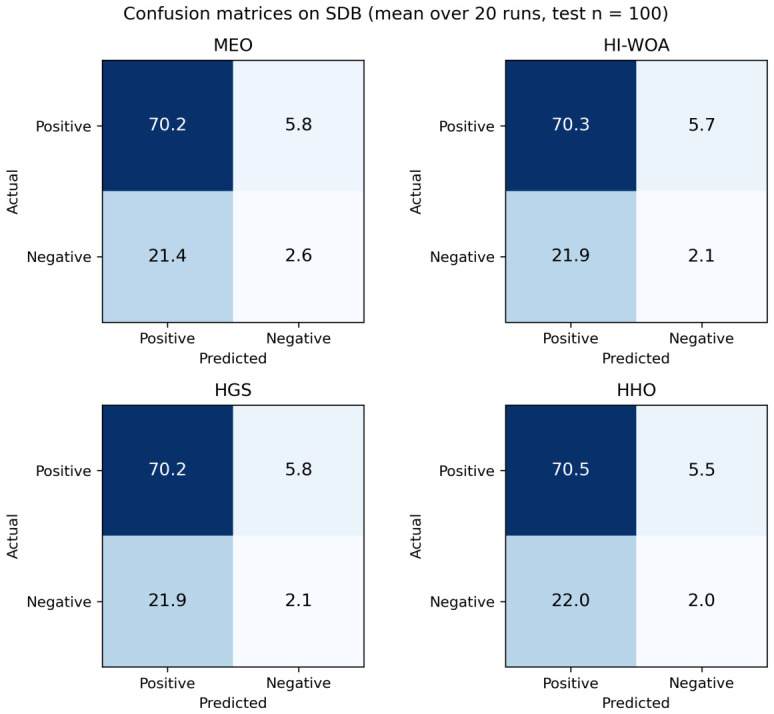
Confusion matrices of the four best optimized ELM models (MEO, HI-WOA, HGS, and HHO) on the SDB dataset, averaged over the 20 runs (test n = 100).

Figure 10 shows the confusion matrices of the four best optimized ELM models on the SDB dataset, averaged over the 20 runs; the very low true-negative counts reflect the strong class imbalance. Table 9 presents the test performance of metaheuristic-optimized ELM models on the SDB dataset. Overall, almost all optimized variants achieve similar or slightly lower accuracy and F1-score than the plain ELM, but several offer modest gains in ROC-AUC. For example, MEO, HGS, GWO, and SeaHO increase AUC from 0.5132 for ELM to about 0.544–0.548 (roughly 6–7% improvement), while their accuracies remain around 0.72–0.73 compared with 0.7410 for ELM. The mean rank values reflect this trade-off: ELM remains among the top methods due to its strong accuracy and F1, whereas MEO and HI-WOA rank well because they balance small drops in accuracy with improved discriminatory power on this highly imbalanced dataset.

**Figure 11:**
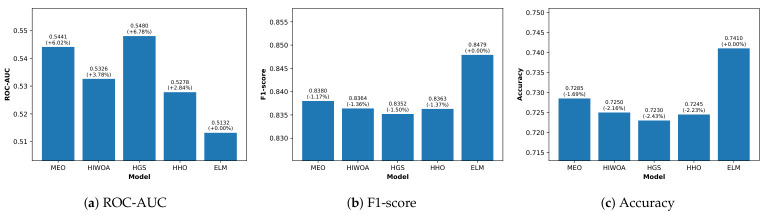
Comparison of the four best-performing metaheuristic-based models (MEO, HIWOA, HGS, HHO) against ELM on the SDB dataset.

For further illustration, Figure 11 focuses on the four best metaheuristic variants on SDB (MEO, HI-WOA, HGS, HHO) and compares them directly with ELM. All four optimizers improved ROC-AUC by approximately 3–7% relative to the standard ELM, but this improvement was accompanied by a 1–2% decrease in accuracy and F1-score. This means that optimization slightly reduces the overall number of correctly classified samples but makes the classifier more sensitive to the minority “no sleep apnea” class, which is reflected in better ranking quality. In a clinical screening context, this trade-off can be acceptable when the priority is to better distinguish risky patients from safe ones rather than maximizing raw accuracy.

**Figure 12:**
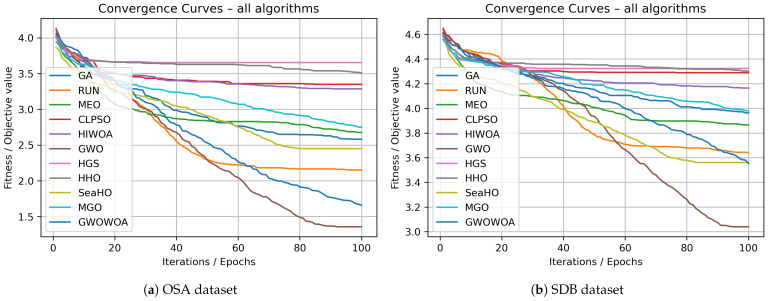
Convergence behavior of the metaheuristic algorithms on the OSA and SDB datasets. Each curve shows the mean fitness value over 20 independent runs across 100 training epochs.

The convergence curves in Figure 12 demonstrate how each metaheuristic reduces the training fitness (cross-entropy loss) over 100 iterations on the OSA and SDB datasets. It is clear that each metaheuristic reduced the training loss, although the rate and stability of convergence differed across algorithms. GWO stands out with a smooth, almost monotonic descent and consistently reaches the lowest final fitness among the methods, while RUN, SeaHO, and MGO also attain strong but slightly higher minima. In contrast, algorithms such as HHO, HGS, CL-PSO, and HI-WOA exhibit early stagnation at relatively high loss values, which suggests weaker exploration and a higher risk of premature convergence to local optima. The curves further indicate that the SDB landscape is more challenging to optimize than the OSA landscape, with higher objective values. These convergence patterns suggest that some metaheuristics optimize the ELM training objective more effectively than others. However, lower training loss did not always correspond to better test ROC-AUC or F1-score. Therefore, convergence behavior should be interpreted as evidence of optimization efficiency, not as direct evidence of clinical or test-set superiority.

Specifically, in our results, GWO often achieves the smallest cross-entropy values on the training data. However, methods such as GA or MGO sometimes obtain similar or better ROC-AUC and F1 on the test set (see Table 8 and Table 9). This happens because the optimized objective is the training loss, while the primary evaluation criteria are based on classification quality, and the dataset, especially SDB, is imbalanced. Very aggressive minimization of loss can push the model toward solutions that fit majority-class patterns or noise, improving the training objective but harming generalization. For this reason, the convergence curves should be read together with the test metrics: they show that the optimizer is working, but the final choice of metaheuristic must be guided by how well the corresponding ELM model performs on unseen cases, not only by how low the training loss becomes.

#### 4.2.3. Computational Cost of Optimization

Table 10 reports the training times of the standard ELM and the metaheuristic-optimized ELM variants on both datasets. The standard ELM required only milliseconds for training, whereas most optimized variants required several seconds, and the heavier optimizers, such as CL-PSO and MGO, required substantially longer training times. Therefore, metaheuristic optimization should be interpreted as an offline tuning procedure rather than a low-cost training method. Its practical value depends on whether the improvement over the standard ELM justifies the additional optimization cost. Once training is complete, however, the optimized ELM retains the low inference cost of a single-hidden-layer model, because prediction only requires a forward pass through the trained network.

#### 4.2.4. Statistical Analysis

To assess whether the differences between the optimized ELM variants and the conventional baselines are statistically significant, we applied a Wilcoxon rank-sum test to the results of the four best optimized ELMs and the five key baselines (SVM, MLP, LR, XGBoost, and the standard ELM), with Benjamini–Hochberg correction for multiple comparisons. The full results are reported in the Appendix B (Table A3 for OSA and Table A4 for SDB). On the OSA dataset, all four optimized ELMs achieved significantly higher ROC-AUC and F1-score than the standard ELM (*p* < 0.05), which confirms that metaheuristic optimization improves the baseline ELM, and they were competitive with the strongest conventional models, SVM and MLP, with no significant difference in ROC-AUC. They also attained significantly higher sensitivity (recall) than SVM and XGBoost, and higher sensitivity than the standard ELM and LR for most variants, while precision (PPV) remained comparable; because sensitivity is the priority for a screening tool, where a missed case is the costliest error, this is a clinically meaningful advantage. On the SDB dataset, optimization did not significantly improve ROC-AUC over the standard ELM and yielded significantly lower accuracy and F1-score than the conventional baselines, reflecting the limited benefit of optimization under strong class imbalance.

### 4.3. Effect of Class-Imbalance Handling on SDB Dataset

When imbalance handling is not applied, the models tend to classify nearly most patients as positive. Sensitivity remains high, ranging from approximately 0.92 to 1.00, while precision stays around 0.76, which is close to the positive-class prevalence. As a result, accuracy, approximately 0.72–0.76, and F1-score, approximately 0.83–0.86, appear relatively strong, as shown in Table 11 and Table 12. However, the threshold-independent metrics provide a much less favorable assessment. MCC ranges from −0.006 to 0.086, indicating performance at or only slightly above random classification. Similarly, ROC-AUC remains between approximately 0.48 and 0.56, which is close to the chance level of 0.50. Although PR-AUC ranges from 0.76 to 0.81, it improves only marginally over the no-skill baseline represented by the positive-class prevalence, approximately 0.762. These results suggest that the apparently high accuracy and F1-score are mainly driven by the models’ tendency to assign most cases to the positive class, rather than by a meaningful ability to distinguish between positive and negative patients. Therefore, under this level of class imbalance, accuracy and F1-score should not be interpreted in isolation, but should be considered together with MCC, ROC-AUC, and PR-AUC.

Imbalance handling appears to shift the models’ operating point rather than improve their underlying discriminative ability. SMOTE, ADASYN, and threshold tuning all reduce accuracy, sensitivity, and F1-score substantially, and these three metrics change significantly for all nine models under each strategy. For the two oversampling methods, the average reductions are approximately 0.15 in accuracy, 0.30 in sensitivity, and 0.14 in F1-score. The reductions are even larger with threshold tuning, reaching approximately 0.22, 0.42, and 0.27, respectively. By contrast, the metrics that summarize discriminative quality show only minor changes. ROC-AUC increases by approximately 0.007 on average with SMOTE and by approximately 0.001 with ADASYN. This change is statistically significant for only one model, SVM, whose AUC increases from about 0.48 to 0.51. PR-AUC changes by less than 0.01 on average and is significant for at most two of the nine models. MCC also remains nearly unchanged, staying within approximately ±0.01, with no consistent direction across models and with significance observed for only one to three models depending on the strategy. Overall, ROC-AUC remains close to 0.50 across all imbalance-handling strategies, while PR-AUC remains between approximately 0.77 and 0.82.

### 4.4. Feature Importance Analysis

#### 4.4.1. Measuring Feature Importance

To examine which predictors drive the models, and whether that predictability rests on diagnostic-derived features, we computed feature importance with two complementary methods, permutation importance and SHAP, for a representative subset of models on each dataset: the standard ELM, the best metaheuristic-optimized ELM (CL-PSO for OSA, HGS for SDB), and the strongest conventional baseline (SVM for OSA, XGBoost for SDB). Permutation importance was computed on the test set across the same 20 stratified splits used in the main experiments. For each split, we recorded the test ROC-AUC, then permuted each feature in turn, ten times, and took the mean resulting drop in ROC-AUC, averaged over the 20 splits; a value near zero means the model does not depend on that feature. SHAP values were obtained with the kernel explainer on a single representative split, using a 50-point summary of the training set as the background, and we report the mean absolute SHAP value per feature. Both methods scored the positive class through the same function, the predicted probability for the conventional classifiers and a softmax of the raw ELM output for the ELM and optimized-ELM models, so the metaheuristic models are treated consistently with the others.

We labeled each predictor as screening-available or PSG/diagnostic-derived. The PSG-derived set comprises the sleep-study outputs: for OSA these are RDI, the apnea–hypopnea indices, the oxygen-saturation and desaturation statistics, the arousal, awakening and PLM indices, total sleep time and sleep efficiency; for SDB, they are AHI, the oxygen-saturation measures, nasal airflow, chest movement and ECG heart rate. This labeling lets us test whether the models rely on quantities that are themselves products of the diagnostic process. The splits use fixed seeds and the permutation and SHAP steps are seeded, so the analysis is reproducible.

#### 4.4.2. Which Predictors Drive the Models

**Figure 13:**
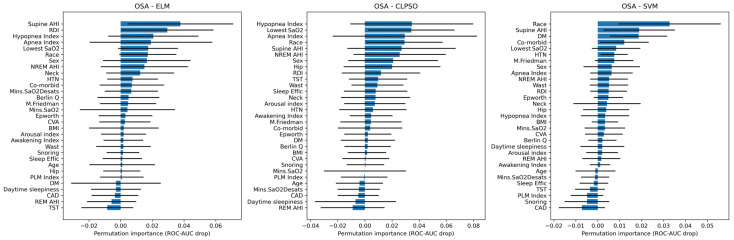
Permutation importance of each predictor on the OSA dataset for the ELM, CL-PSO-ELM, and SVM models. Blue bars represent the mean drop in test ROC-AUC after permuting each feature, averaged over 20 stratified runs with 10 permutations per run; black horizontal lines denote ±1 standard deviation across the 20 runs. Values near or below zero indicate limited or unstable contribution to model discrimination.

**Figure 14:**
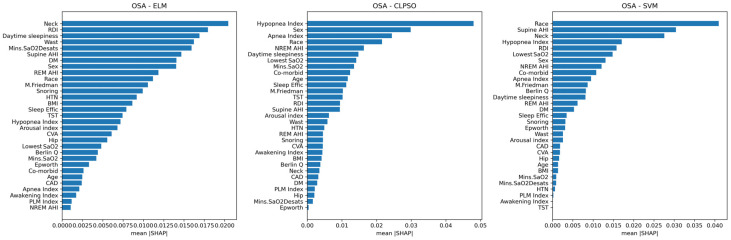
Mean absolute SHAP value of each predictor on OSA, for the same three models.

On OSA, the models are reasonably discriminative (test ROC-AUC 0.69 to 0.75), and a consistent pattern emerges (as shown in Figure 13 and Figure 14). Under permutation importance, averaged over the 20 runs, the leading predictors for the standard ELM and the optimized CL-PSO-ELM are dominated by polysomnographic indices: the apnea–hypopnea family (Supine, NREM, Apnea and Hypopnea indices), RDI, and the lowest oxygen saturation fill most of the top positions. SHAP, computed on a single split, shows the same polysomnography-heavy ranking for the CL-PSO-ELM; for the plain ELM, it agrees that RDI, an oxygen-desaturation measure and Supine AHI are influential, but it also ranks anthropometric and questionnaire features, such as neck circumference and daytime sleepiness, among the most important. The SVM, the strongest baseline, relies somewhat more on demographics, with Race its single most important feature by both methods, although Supine AHI is its second. Across the three models, several of the strongest predictors remain PSG-derived quantities.

**Figure 15:**
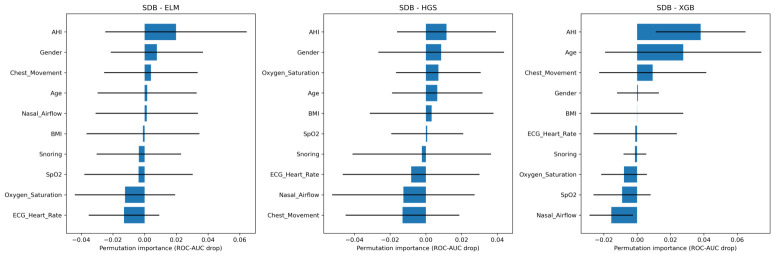
Permutation importance (mean drop in test ROC-AUC over 20 runs) of each predictor on SDB, for the ELM, the HGS-ELM, and XGBoost.

**Figure 16:**
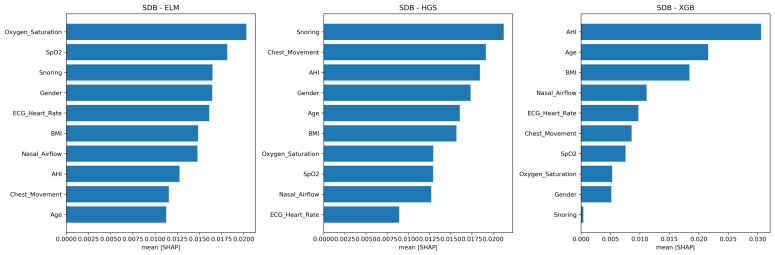
Mean absolute SHAP value of each predictor on SDB, for the same three models.

On SDB, the models discriminate weakly (test ROC-AUC 0.51 to 0.56), so the importance values are small and, for the two near-chance ELM models, somewhat unstable between the two methods (see Figure 15 and Figure 16). The clearest and most consistent signal comes from XGBoost, the only model with ROC-AUC clearly above chance (0.555): permutation importance and SHAP both rank AHI first and Age second by a wide margin. AHI is also the top permutation feature for the standard ELM and for the optimized HGS-ELM. Among the screening-available predictors, only Age and Gender carry appreciable weight, while BMI and Snoring contribute little. AHI is a sleep-apnea severity index, that is, a direct product of the diagnostic process, so the limited predictability that the SDB models do achieve rests largely on a diagnostic-derived feature rather than on the simple screening variables.

The two methods agree on the leading features for the more discriminative models: the Hypopnea Index for CL-PSO and Race for SVM on OSA, and AHI and Age for XGBoost on SDB, each rank first under both permutation importance and SHAP. They diverge mainly for the near-chance SDB ELM and HGS models, which is expected when there is little stable signal to attribute. Because the two methods quantify different things, a drop in ROC-AUC versus an average marginal contribution to the predicted probability, exact rank agreement is not expected, and the consistency at the top of the discriminative models is the meaningful result.

## 5. Discussion

The results should be interpreted in terms of three connected issues: predictive performance, generalization under limited sample sizes, and the additional offline cost introduced by metaheuristic optimization. Taken together, the results across both datasets reveal a recurring trade-off between predictive performance, generalization, and computational cost. On the OSA dataset, SVM and MLP delivered the strongest test ROC-AUC and F1-scores among the baseline models. However, their high training scores and larger train–test gaps suggest possible overfitting in a relatively small clinical sample. LR and the plain ELM showed slightly lower test performance, but their smaller generalization gaps indicate more stable behavior across repeated splits.

The SDB dataset presented a different challenge because of its strong class imbalance. Accuracy, recall, and F1-score appeared relatively high for several models, but ROC-AUC remained modest. This indicates that high aggregate scores may partly reflect a tendency to favor the majority positive class rather than reliable discrimination between SDB and non-SDB cases. Therefore, the SDB results should be interpreted cautiously, especially for clinical screening settings where sensitivity, specificity, and threshold-dependent decisions are important.

The dedicated imbalance-handling analysis (Section 4.3) makes this point concrete: SMOTE, ADASYN, and decision-threshold tuning correct a reporting artifact rather than improving the classifier. The artifact is the majority-class bias that inflates accuracy and F1; once it is removed, the weak underlying signal is exposed, with MCC and ROC-AUC sitting near chance for every classifier and both ELM optimizers. Resampling and thresholding redistribute a fixed amount of skill rather than adding information, so the limited signal must lie in the structured SDB predictors themselves and not in the class balance. The SDB feature set therefore supports cautious screening rather than definitive discrimination Accordingly, the present SDB results should be regarded as a proof of concept for metaheuristic-optimized ELM screening rather than evidence of a clinically deployable diagnostic tool, and the near-chance ROC-AUC and MCC values indicate that further model refinement and external validation are needed to improve discriminative performance.

Metaheuristic optimization improved the ELM results more clearly on the OSA dataset than on the SDB dataset. On OSA, MGO, RUN, and CL-PSO improved the standard ELM in accuracy, F1-score, and ROC-AUC. These gains suggest that optimizing the hidden-layer weights and biases can reduce the limitations of random ELM initialization. However, the optimized ELM models did not uniformly outperform all baseline classifiers. SVM achieved the highest ROC-AUC, indicating stronger ranking-based discrimination, whereas several optimized ELM variants achieved higher recall and F1-score, and CL-PSO achieved higher accuracy. Thus, the advantage of optimized ELM should be framed as competitive threshold-dependent performance with low inference cost, not as overall superiority over SVM.

On the SDB dataset, the benefits of optimization were more limited. Several optimized ELM variants produced modest ROC-AUC improvements, but these gains were accompanied by small reductions in accuracy and F1-score. This pattern suggests that optimization may have shifted the model toward better discrimination of the minority class, but the strong imbalance and limited sample size constrained the overall benefit. The convergence curves support this interpretation: lower training loss did not always correspond to better test performance, indicating that the training objective should not be interpreted alone. This distinction is important because the optimization process minimizes a training loss, whereas screening performance depends on discrimination, threshold behavior, and generalization to unseen patients.

From a computational perspective, metaheuristic optimization introduces a clear offline training cost. The standard ELM trains within milliseconds, whereas optimized ELM variants require several seconds or longer depending on the optimizer. This additional cost is not justified by training efficiency alone. It is justified only when the optimized model provides a useful gain in discrimination, threshold-dependent performance, robustness, or deployment simplicity compared with the standard ELM and competing baselines. This is important because SVM and other conventional classifiers achieved similar or better performance on some metrics. Once training is complete, however, the optimized ELM retains the low inference cost of the standard ELM, which remains relevant for lightweight screening applications.

The feature-importance analysis (Section 4.4) helps in understanding how the reported performance should be interpreted. The predictors the models rely on most are largly outputs of a sleep study: the apnea–hypopnea indices, RDI and oxygen-desaturation measures on OSA, and AHI on SDB. Because these quantities are produced by the same diagnostic process the models are meant to anticipate, their prominence indicates that the reported performance partly reflects access to diagnostic information rather than genuine early-screening ability. This motivates a screening-only analysis that removes the PSG-derived predictors and re-evaluates the models on the demographic and questionnaire features available before a sleep study.

### Limitations

This study has few limitations that should be acknowledged when interpreting the results:

- The experiments rely on two datasets from a specific clinical context, each with a relatively small sample size. Accordingly, the generality of the conclusions to other populations or acquisition protocols is not guaranteed, and real-world data from electronic health records, which are typically noisier and less complete, would require recalibration and external validation before clinical deployment.
- Model interpretability was examined through permutation importance and SHAP, which identified the dominant predictors; a deeper, clinically oriented interpretability analysis and prospective validation remain for future work.
- The metaheuristic algorithms, the ELM architecture, and the investigated ML models were configured using reasonable but fixed hyperparameter settings. More exhaustive hyperparameter tuning could further improve performance or change the relative rankings of the methods.

## 6. Conclusions and Future Work

This study investigated a lightweight, metaheuristic-optimized ELM framework for early diagnosis of OSA and SDB using demographic/clinical predictors. After standardizing features with a strict train–test separation, we evaluated eight baseline classifiers and then optimized the ELM hidden-layer weights and biases using eleven metaheuristic algorithms. On the OSA dataset, baseline results showed that SVM and MLP achieved the strongest overall discrimination among conventional models (e.g., SVM ROC-AUC = 0.7462, MLP ROC-AUC = 0.7263). At the same time, the plain ELM provided competitive but lower test performance (accuracy = 0.6573, F1 = 0.6153, ROC-AUC = 0.6527). Importantly, metaheuristic tuning improved the standard ELM on the OSA dataset. For example, CL-PSO increased test ROC-AUC to 0.7329 and accuracy to 0.7000, while RUN improved F1-score to 0.6651 compared with 0.6153 for the standard ELM. These gains indicate that optimizing the hidden-layer parameters can improve ELM performance. However, the optimized ELM variants did not uniformly outperform all baseline classifiers. SVM achieved the highest ROC-AUC, whereas several optimized ELM variants achieved stronger recall and F1-score, and CL-PSO achieved higher accuracy. Therefore, the optimized ELM models should be interpreted as competitive screening-oriented decision-support models rather than standalone or clearly superior diagnostic tools.

The SDB dataset, with its heavy class skew, told a different story. Baseline models produced high accuracy and F1 figures but only modest ROC-AUC values, and the plain ELM ranked among the stronger baselines on accuracy and F1 (0.7410 and 0.8479, respectively) while its ROC-AUC remained limited at 0.5132. Here, metaheuristic optimization mostly shifted the balance toward better discrimination rather than higher aggregate scores. HGS, for example, lifted ROC-AUC to 0.5480 and MEO to 0.5441, even as accuracy slipped a little (MEO at 0.7285 against 0.7410 for the plain ELM). These findings indicate that, under severe imbalance, optimization may improve ranking ability modestly, but the gain in ROC-AUC may come at the cost of lower accuracy and F1-score. The timing analysis adds an important practical qualification. Optimization is best treated as an offline step: training the standard ELM takes around 0.0046 s on OSA, whereas the optimized variants require roughly 10–20 s for most algorithms and up to 40–56 s for the heavier optimizers. This additional cost should be weighed against the observed performance gains, especially when conventional baselines such as SVM perform similarly or better on some metrics. After optimization, however, inference remains inexpensive because the final ELM model uses a fixed single-hidden-layer structure.

Future work should focus on improving clinical readiness and generalization. First, broader validation is needed across larger, more diverse cohorts (including external sites) to confirm robustness across populations and acquisition settings, especially given the limited sample sizes used here. Second, although we evaluated resampling (SMOTE and ADASYN) and decision-threshold tuning on the SDB set, these rebalanced the operating point without improving ranking; stronger imbalance-aware learning, such as cost-sensitive objectives or thresholds tied to a clinical operating point, is still worth pursuing. Third, building on the permutation-importance and SHAP analyses reported here, deeper interpretability and verification that the optimized-ELM decisions align with medical knowledge should be pursued, including the screening-only evaluation motivated by the prominence of polysomnographic predictors. Fourth, a more systematic exploration of hyperparameters (ELM architecture choices and optimizer settings) and potentially multi-objective optimization (optimizing AUC/F1 and calibration jointly, rather than only the training loss) may further improve the ranking and consistency of the optimized model.

## Figures and Tables

**Table 1:** Summary of the two datasets used in this study.

Dataset	No. Samples	No. Features	Negative Samples	Positive Samples	Positive Rate (%)
OSA	274	31	149	125	45.6
SDB	500	10	119	381	76.2

**Table 2:** Overview of predictor variables across the OSA and SDB datasets.

Feature	Type	In OSA	In SDB	Description
Race	Categorical	Yes	No	Race/ethnic group category.
Age	Numeric (continuous)	Yes	Yes	Patient age in years.
Sex	Categorical (binary)	Yes	Yes	Patient sex.
BMI *	Numeric/categorical	Yes	Yes	Body mass index or BMI category.
Epworth	Numeric (ordinal)	Yes	No	Epworth Sleepiness Scale total score.
Waist	Numeric (continuous)	Yes	No	Waist circumference (cm).
Hip	Numeric (continuous)	Yes	No	Hip circumference (cm).
RDI	Numeric (continuous)	Yes	No	Respiratory Disturbance Index per hour.
Neck	Numeric (continuous)	Yes	No	Neck circumference (cm).
M.Friedman	Ordinal categorical	Yes	No	Friedman tongue position grade (1–4).
Co-morbid	Categorical	Yes	No	Presence or count of comorbidities.
Snoring	Categorical (binary)	Yes	Yes	Snoring indicator.
Daytime sleepiness	Categorical (binary)	Yes	No	Self-reported daytime sleepiness.
DM	Categorical (binary)	Yes	No	Diabetes mellitus status.
HTN	Categorical (binary)	Yes	No	Hypertension status.
CAD	Categorical (binary)	Yes	No	Coronary artery disease status.
CVA	Categorical (binary)	Yes	No	History of cerebrovascular accident.
TST	Numeric (continuous)	Yes	No	Total sleep time.
Sleep Effic	Numeric (continuous)	Yes	No	Sleep efficiency (%).
REM AHI	Numeric (continuous)	Yes	No	Apnea–Hypopnea Index in REM sleep.
NREM AHI	Numeric (continuous)	Yes	No	Apnea–Hypopnea Index in NREM sleep.
Supine AHI	Numeric (continuous)	Yes	No	Apnea–Hypopnea Index in supine position.
Apnea Index	Numeric (continuous)	Yes	No	Number of apnea events per hour.
Hypopnea Index	Numeric (continuous)	Yes	No	Number of hypopnea events per hour.
Berlin Q	Categorical	Yes	No	Berlin questionnaire risk category.
Arousal index	Numeric (continuous)	Yes	No	Number of arousals per hour.
Awakening Index	Numeric (continuous)	Yes	No	Number of awakenings per hour.
PLM Index	Numeric (continuous)	Yes	No	Periodic limb movement index per hour.
Mins.SaO_2_	Numeric (continuous)	Yes	No	Minutes below oxygen saturation threshold.
Mins.SaO_2_Desats	Numeric (continuous)	Yes	No	Minutes with oxygen desaturation events.
Lowest SaO_2_	Numeric (continuous)	Yes	No	Lowest oxygen saturation recorded.
Oxygen_Saturation	Numeric (continuous)	No	Yes	Average oxygen saturation during recording.
AHI	Numeric (continuous)	No	Yes	Overall Apnea–Hypopnea Index.
ECG_Heart_Rate	Numeric (continuous)	No	Yes	Heart rate derived from ECG.
SpO_2_	Numeric (continuous)	No	Yes	Average peripheral oxygen saturation (%).
Nasal_Airflow	Numeric (continuous)	No	Yes	Normalized nasal airflow signal.
Chest_Movement	Numeric (continuous)	No	Yes	Normalized chest movement signal.

* BMI is treated as a categorical variable in the OSA dataset and as a numeric variable in the SDB dataset.

**Table 3:** Baseline classifiers and main hyperparameters used in the experiments.

Model	Key Hyperparameters
ELM	layer_sizes = 50; act_name = relu
LR (LogReg)	max_iter = 200
RF	n_estimators = 20
SVM (RBF)	kernel = rbf; probability = True
XGB	n_estimators = 20; max_depth = 4; learning_rate = 0.05;
	subsample = 0.8; colsample_bytree = 0.8;
	eval_metric = logloss
MLP	hidden_layer_sizes = 100; activation = relu; solver = adam;
	max_iter = 200
KNN	n_neighbors = 5; weights = distance; metric = minkowski
DT	max_depth = None

**Table 4:** Test-set classification performance of baseline models on the OSA dataset. Mean rank is the average ranking of each model across all evaluation metrics, with lower values indicating better overall performance.

Model	Measure	Accuracy	Precision	Recall	F1-Score	ROC-AUC	Mean Rank
DT	Avg	0.5736	0.5345	0.5220	0.5255	0.5693	8.0
	Std	0.0564	0.0684	0.0794	0.0651	0.0552	
ELM	Avg	0.6573	0.6332	0.6020	0.6153	0.6527	4.4
	Std	0.0628	0.0751	0.0680	0.0620	0.0616	
KNN	Avg	0.6355	0.6175	0.5340	0.5707	0.7096	6.4
	Std	0.0603	0.0865	0.0749	0.0713	0.0584	
LR	Avg	0.6582	0.6331	0.6040	0.6155	0.7209	3.2
	Std	0.0615	0.0720	0.0850	0.0664	0.0653	
MLP	Avg	0.6600	0.6334	**0.6160**	**0.6210**	0.7263	**2.0**
	Std	0.0749	0.0943	0.1025	0.0847	0.0561	
RF	Avg	0.6564	0.6423	0.5740	0.6010	0.6885	4.4
	Std	0.0472	0.0785	0.0902	0.0593	0.0611	
SVM	Avg	**0.6836**	**0.6973**	0.5440	0.6063	**0.7462**	2.6
	Std	0.0470	0.0758	0.1017	0.0736	0.0633	
XGB	Avg	0.6536	0.6393	0.5600	0.5920	0.6886	5.0
	Std	0.0423	0.0609	0.0954	0.0624	0.0460	

**Table 5:** Training and test performance of baseline classifiers on the OSA dataset. It presents the generalization gap (Δ) for accuracy, F1-score, and ROC-AUC, where Δ = train − test.

Model	Accuracy			F1-Score			roc_auc		
	Train	Test	Δ	Train	Test	Δ	Train	Test	Δ
DT	1.0000	0.5736	0.4264	1.0000	0.5255	0.4745	1.0000	0.5693	0.4307
ELM	0.7740	0.6573	0.1167	0.7435	0.6153	0.1282	0.7696	0.6527	0.1169
KNN	1.0000	0.6355	0.3645	1.0000	0.5707	0.4293	1.0000	0.7096	0.2904
LR	0.7521	0.6582	0.0939	0.7142	0.6155	0.0987	0.8402	0.7209	0.1193
MLP	0.9553	0.6600	0.2953	0.9509	0.6210	0.3299	0.9923	0.7263	0.2660
RF	0.9966	0.6564	0.3402	0.9962	0.6010	0.3952	0.9999	0.6885	0.3114
SVM	0.8543	0.6836	0.1707	0.8282	0.6063	0.2219	0.9364	0.7462	0.1902
XGB	0.8879	0.6536	0.2343	0.8719	0.5920	0.2799	0.9627	0.6886	0.2741

**Table 6:** Training and testing times (in seconds) of baseline classifiers on the OSA dataset; mean rank indicates the average efficiency ranking across models (lower values mean faster overall performance).

Model	Measure	Training Time	Testing Time	Mean Rank
DT	Avg	0.0063	0.0005	2.0
	Std	0.0019	0.0003	
ELM	Avg	**0.0046**	**0.0001**	**1.0**
	Std	0.0054	0.0001	
KNN	Avg	0.1295	0.0051	6.5
	Std	0.5372	0.0013	
LR	Avg	0.0076	0.0006	3.0
	Std	0.0037	0.0004	
MLP	Avg	0.3751	0.0009	6.0
	Std	0.0939	0.0004	
RF	Avg	0.0622	0.0066	6.5
	Std	0.0218	0.0027	
SVM	Avg	0.0507	0.0070	6.5
	Std	0.0178	0.0033	
XGB	Avg	0.0286	0.0014	4.5
	Std	0.0065	0.0006	

**Table 7:** Test classification performance of baseline models on the SDB dataset.

Model	Measure	Accuracy	Precision	Recall	F1-Score	ROC-AUC	Mean Rank
DT	Avg	0.6345	0.7695	0.7408	0.7542	0.5194	6.4
	Std	0.0467	0.0258	0.0544	0.0369	0.0521	
ELM	Avg	0.7410	0.7650	0.9513	0.8479	0.5132	4.8
	Std	0.0295	0.0116	0.0355	0.0194	0.0301	
KNN	Avg	0.7190	0.7629	0.9145	0.8317	0.5426	5.6
	Std	0.0279	0.0142	0.0275	0.0176	0.0508	
LR	Avg	0.7590	0.7598	0.9987	0.8630	0.5380	4.2
	Std	0.0031	0.0007	0.0040	0.0020	0.0527	
MLP	Avg	0.7460	**0.7697**	0.9500	0.8502	0.5430	3.2
	Std	0.0237	0.0094	0.0327	0.0160	0.0582	
RF	Avg	0.7300	0.7625	0.9368	0.8405	0.5329	5.6
	Std	0.0192	0.0104	0.0288	0.0130	0.0532	
SVM	Avg	**0.7600**	0.7600	**1.0000**	**0.8636**	0.4855	3.4
	Std	0.0000	0.0000	0.0000	0.0000	0.0568	
XGB	Avg	**0.7600**	0.7600	**1.0000**	**0.8636**	**0.5804**	**2.0**
	Std	0.0000	0.0000	0.0000	0.0000	0.0511	

**Table 8:** Test classification performance of metaheuristic-optimized ELM models on the OSA dataset. ELM denotes the baseline (non-optimized) version.

Model	Measure	Accuracy	Precision	Recall	F1-Score	ROC-AUC	Mean Rank
CLPSO	Avg	**0.7000**	**0.6798**	0.6420	0.6582	**0.7329**	3.0
	Std	0.0423	0.0457	0.0904	0.0602	0.0382	
GA	Avg	0.6873	0.6541	**0.6740**	0.6611	0.7157	4.0
	Std	0.0415	0.0513	0.0771	0.0462	0.0408	
GWO	Avg	0.6818	0.6500	0.6560	0.6515	0.7164	5.8
	Std	0.0492	0.0561	0.0716	0.0552	0.0519	
GWOWOA	Avg	0.6827	0.6573	0.6460	0.6483	0.7223	4.8
	Std	0.0406	0.0556	0.0737	0.0446	0.0381	
HGS	Avg	0.6818	0.6533	0.6480	0.6468	0.7074	6.6
	Std	0.0499	0.0561	0.0968	0.0650	0.0485	
HHO	Avg	0.6745	0.6464	0.6300	0.6343	0.7066	9.6
	Std	0.0627	0.0687	0.1153	0.0812	0.0505	
HIWOA	Avg	0.6573	0.6304	0.5960	0.6112	0.6839	11.6
	Std	0.0597	0.0705	0.0860	0.0733	0.0550	
MEO	Avg	0.6755	0.6506	0.6220	0.6333	0.7214	8.0
	Std	0.0418	0.0528	0.0846	0.0589	0.0559	
MGO	Avg	0.6955	0.6689	0.6640	0.6639	0.7286	**2.2**
	Std	0.0368	0.0497	0.0679	0.0419	0.0478	
RUN	Avg	0.6927	0.6590	**0.6740**	**0.6651**	0.7187	2.6
	Std	0.0504	0.0547	0.0771	0.0581	0.0539	
SeaHO	Avg	0.6791	0.6486	0.6440	0.6445	0.7111	8.0
	Std	0.0614	0.0726	0.0917	0.0744	0.0594	
ELM	Avg	0.6573	0.6332	0.6020	0.6153	0.6527	11.2
	Std	0.0628	0.0751	0.0680	0.0620	0.0616	

**Table 9:** Test classification performance of metaheuristic-optimized ELM models on the SDB dataset.

Model	Measure	Accuracy	Precision	Recall	F1-Score	ROC-AUC	Mean Rank
CLPSO	Avg	0.7175	0.7579	0.9230	0.8323	0.4985	8.6
	Std	0.0183	0.0077	0.0215	0.0121	0.0516	
GA	Avg	0.7140	0.7649	0.9007	0.8271	0.5300	8.2
	Std	0.0190	0.0095	0.0251	0.0130	0.0571	
GWO	Avg	0.7120	0.7673	0.8914	0.8246	0.5454	7.2
	Std	0.0253	0.0134	0.0280	0.0168	0.0518	
GWOWOA	Avg	0.7180	**0.7681**	0.9013	0.8293	0.5404	6.0
	Std	0.0164	0.0106	0.0184	0.0104	0.0506	
HGS	Avg	0.7230	0.7624	0.9237	0.8352	**0.5480**	5.0
	Std	0.0187	0.0118	0.0207	0.0116	0.0493	
HHO	Avg	0.7245	0.7620	0.9270	0.8363	0.5278	5.6
	Std	0.0295	0.0143	0.0306	0.0189	0.0722	
HIWOA	Avg	0.7250	0.7631	0.9257	0.8364	0.5326	4.6
	Std	0.0164	0.0097	0.0231	0.0109	0.0447	
MEO	Avg	0.7285	0.7666	0.9243	0.8380	0.5441	**3.0**
	Std	0.0278	0.0152	0.0276	0.0174	0.0647	
MGO	Avg	0.6935	0.7545	0.8842	0.8139	0.5094	11.6
	Std	0.0303	0.0112	0.0404	0.0216	0.0667	
RUN	Avg	0.7165	0.7635	0.9086	0.8296	0.5215	8.0
	Std	0.0320	0.0174	0.0294	0.0201	0.0803	
SeaHO	Avg	0.7205	0.7648	0.9132	0.8323	0.5450	5.6
	Std	0.0190	0.0092	0.0223	0.0126	0.0329	
ELM	Avg	**0.7410**	0.7650	**0.9513**	**0.8479**	0.5132	3.4
	Std	0.0295	0.0116	0.0355	0.0194	0.0301	

**Table 10:** Training times (in seconds) of baseline ELM and metaheuristic-based ELM versions on OSA and SDB datasets.

Model	Measure	OSA	SDB
CLPSO	Avg	56.7140	30.8554
	Std	1.4979	3.4613
GA	Avg	12.2153	13.8607
	Std	0.3092	3.4478
GWO	Avg	11.4065	10.5191
	Std	0.2875	0.3267
GWOWOA	Avg	11.4524	14.6931
	Std	0.5361	2.7607
HGS	Avg	10.6527	10.5610
	Std	0.5867	0.9460
HHO	Avg	18.6361	18.7242
	Std	0.7000	1.3378
HIWOA	Avg	12.9272	12.9110
	Std	0.8009	0.7845
MEO	Avg	17.5411	22.7018
	Std	0.5319	4.3979
MGO	Avg	40.1385	46.5233
	Std	1.1942	4.0455
RUN	Avg	20.2531	24.6024
	Std	0.7883	9.1322
SeaHO	Avg	17.4395	17.7751
	Std	0.5797	2.9496
ELM	Avg	**0.0046**	**0.0911**
	Std	0.0054	0.2988

**Table 11:** Test-set performance of the baseline classifiers on the SDB dataset under no handling and the three imbalance-handling strategies (SMOTE, ADASYN, and decision-threshold tuning). Values are means over 20 stratified runs. The no-handling rows correspond to the no-resampling baseline; their classification metrics (accuracy, precision, recall, F1) are reported in Table 7.

Model	Method	Accuracy	Precision (PPV)	Sensitivity (Recall)	F1-Score	ROC-AUC	PR-AUC	MCC
**SVM**	None	0.760	0.760	1.000	0.864	0.476	0.755	0.000
	SMOTE	0.639	0.759	0.770	0.764	0.510	0.785	−0.004
	ADASYN	0.637	0.760	0.763	0.761	0.521	0.786	0.001
	Threshold	0.536	0.700	0.581	0.589	0.476	0.755	−0.032
**MLP**	None	0.745	0.769	0.949	0.850	0.545	0.796	0.086
	SMOTE	0.623	0.772	0.716	0.742	0.536	0.792	0.042
	ADASYN	0.646	0.777	0.752	0.763	0.548	0.800	0.061
	Threshold	0.461	0.793	0.402	0.492	0.545	0.796	0.048
**XGB**	None	0.760	0.760	1.000	0.864	0.580	0.808	0.000
	SMOTE	0.593	0.791	0.631	0.700	0.573	0.822	0.090
	ADASYN	0.588	0.787	0.629	0.696	0.570	0.817	0.077
	Threshold	0.499	0.793	0.474	0.563	0.555	0.808	0.052
**LR**	None	0.759	0.760	0.999	0.863	0.538	0.792	−0.006
	SMOTE	0.566	0.787	0.589	0.673	0.537	0.792	0.070
	ADASYN	0.570	0.782	0.603	0.680	0.535	0.791	0.059
	Threshold	0.514	0.718	0.505	0.563	0.538	0.792	0.052
**ELM**	None	0.741	0.765	0.951	0.848	0.514	0.780	0.059
	SMOTE	0.572	0.757	0.643	0.694	0.509	0.781	−0.010
	ADASYN	0.563	0.755	0.629	0.685	0.514	0.783	−0.015
	Threshold	0.549	0.780	0.577	0.605	0.514	0.780	0.050

**Table 12:** Test-set performance of the metaheuristic-optimized ELM models on the SDB dataset under the same conditions, with the standard ELM included as the non-optimized reference. Values are means over 20 stratified runs.

Model	Method	Accuracy	Precision (PPV)	Sensitivity (Recall)	F1-Score	ROC-AUC	PR-AUC	MCC
**ELM**	None	0.741	0.765	0.951	0.848	0.514	0.780	0.059
	SMOTE	0.572	0.757	0.643	0.694	0.509	0.781	−0.010
	ADASYN	0.563	0.755	0.629	0.685	0.514	0.783	−0.015
	Threshold	0.549	0.780	0.577	0.605	0.514	0.780	0.050
**MEO**	None	0.722	0.765	0.915	0.833	0.544	0.782	0.032
	SMOTE	0.569	0.758	0.636	0.691	0.514	0.785	−0.007
	ADASYN	0.580	0.765	0.645	0.699	0.525	0.789	0.014
	Threshold	0.529	0.759	0.552	0.596	0.520	0.782	0.008
**HIWOA**	None	0.733	0.766	0.934	0.841	0.522	0.785	0.048
	SMOTE	0.581	0.770	0.639	0.697	0.533	0.794	0.030
	ADASYN	0.575	0.757	0.650	0.699	0.486	0.766	−0.012
	Threshold	0.534	0.742	0.559	0.564	0.522	0.785	0.029
**HGS**	None	0.725	0.761	0.930	0.837	0.548	0.772	0.003
	SMOTE	0.580	0.773	0.635	0.695	0.531	0.797	0.036
	ADASYN	0.581	0.762	0.653	0.703	0.519	0.780	0.007
	Threshold	0.497	0.752	0.489	0.547	0.517	0.772	0.007
**HHO**	None	0.738	0.765	0.944	0.845	0.527	0.776	0.048
	SMOTE	0.575	0.771	0.626	0.690	0.533	0.791	0.032
	ADASYN	0.569	0.757	0.638	0.691	0.506	0.777	−0.014
	Threshold	0.587	0.763	0.660	0.676	0.527	0.776	0.026

## Data Availability

The Sleep-Disordered Breathing (SDB) dataset used in this study is publicly available on Kaggle at https://www.kaggle.com/datasets/ziya07/sleep-disordered-breathing-detection (accessed on 13 December 2025). The processed Obstructive Sleep Apnea (OSA) dataset is derived from a previously published study [39] and is available from the corresponding author upon reasonable request, subject to applicable data-sharing restrictions. The source code used to run the experiments is publicly available at https://github.com/Thaer83/metaheuristic-elm-sdb-screening (accessed on 13 December 2025) (version 1.0.0).

## Appendix A. Training-Set Performance of the Baseline Classifiers

**Table A1:** Training-set classification performance of baseline models on the OSA dataset.

Model	Measure	Accuracy	Precision	Recall	F1	roc_auc	Mean Rank
DT	Avg	**1.0000**	**1.0000**	**1.0000**	**1.0000**	**1.0000**	1.0
	Std	0.0000	0.0000	0.0000	0.0000	0.0000	
ELM	Avg	0.7740	0.7708	0.7195	0.7435	0.7696	7.2
	Std	0.0218	0.0239	0.0454	0.0293	0.0230	
KNN	Avg	**1.0000**	**1.0000**	**1.0000**	**1.0000**	**1.0000**	1.0
	Std	0.0000	0.0000	0.0000	0.0000	0.0000	
LR	Avg	0.7521	0.7536	0.6790	0.7142	0.8402	7.8
	Std	0.0133	0.0151	0.0229	0.0173	0.0108	
MLP	Avg	0.9553	0.9516	0.9505	0.9509	0.9923	4.0
	Std	0.0100	0.0089	0.0224	0.0115	0.0024	
RF	Avg	0.9966	0.9975	0.9950	0.9962	0.9999	3.0
	Std	0.0036	0.0044	0.0061	0.0039	0.0002	
SVM	Avg	0.8543	0.8959	0.7710	0.8282	0.9364	6.0
	Std	0.0164	0.0172	0.0377	0.0224	0.0072	
XGB	Avg	0.8879	0.9112	0.8365	0.8719	0.9627	5.0
	Std	0.0152	0.0224	0.0268	0.0178	0.0057	

**Table A2:** Training-set classification performance of baseline models on the SDB dataset.

Model	Measure	Accuracy	Precision	Recall	F1	roc_auc	Mean Rank
DT	Avg	**1.0000**	**1.0000**	**1.0000**	**1.0000**	**1.0000**	1.0
	Std	0.0000	0.0000	0.0000	0.0000	0.0000	
ELM	Avg	0.7795	0.7860	0.9770	0.8711	0.5612	7.0
	Std	0.0101	0.0089	0.0088	0.0053	0.0226	
KNN	Avg	**1.0000**	**1.0000**	**1.0000**	**1.0000**	**1.0000**	1.0
	Std	0.0000	0.0000	0.0000	0.0000	0.0000	
LR	Avg	0.7621	0.7624	0.9995	0.8650	0.6098	6.0
	Std	0.0009	0.0002	0.0012	0.0006	0.0136	
MLP	Avg	0.8208	0.8172	0.9856	0.8935	0.8698	5.0
	Std	0.0090	0.0098	0.0065	0.0046	0.0124	
RF	Avg	0.9965	0.9958	0.9997	0.9977	1.0000	1.0
	Std	0.0024	0.0028	0.0015	0.0015	0.0001	
SVM	Avg	0.7656	0.7649	**1.0000**	0.8668	0.5389	8.0
	Std	0.0034	0.0026	0.0000	0.0017	0.4601	
XGB	Avg	0.7626	0.7626	**1.0000**	0.8653	0.9324	4.0
	Std	0.0006	0.0004	0.0000	0.0003	0.0106	

## Appendix B. Statistical Comparison of Optimized ELM Variants and Baselines

**Table A3:** Statistical comparison on the OSA dataset between the four best optimized ELM models and key baseline classifiers. Each value reports the mean-metric difference between the optimized ELM and the corresponding baseline. Positive values favor the optimized ELM. Asterisks indicate Benjamini–Hochberg adjusted p<0.05 based on the Wilcoxon rank-sum test.

**ROC-AUC**					
**Optimized**	**SVM**	**MLP**	**LR**	**XGB**	**ELM**
**MGO**	−0.018	+0.002	+0.008	+0.040	+0.076 *
**RUN**	−0.028	−0.008	−0.002	+0.030	+0.066 *
**CLPSO**	−0.013	+0.007	+0.012	+0.044 *	+0.080 *
**GA**	−0.030	−0.011	−0.005	+0.027	+0.063 *
**F1-Score**					
**Optimized**	**SVM**	**MLP**	**LR**	**XGB**	**ELM**
**MGO**	+0.058 *	+0.043	+0.048 *	+0.072 *	+0.049 *
**RUN**	+0.059 *	+0.044	+0.050 *	+0.073 *	+0.050 *
**CLPSO**	+0.052	+0.037	+0.043	+0.066 *	+0.043 *
**GA**	+0.055 *	+0.040	+0.046 *	+0.069 *	+0.046 *
**Sensitivity (Recall)**					
**Optimized**	**SVM**	**MLP**	**LR**	**XGB**	**ELM**
**MGO**	+0.120 *	+0.048	+0.060 *	+0.104 *	+0.062 *
**RUN**	+0.130 *	+0.058	+0.070 *	+0.114 *	+0.072 *
**CLPSO**	+0.098 *	+0.026	+0.038	+0.082 *	+0.040
**GA**	+0.130 *	+0.058	+0.070 *	+0.114 *	+0.072 *
**Precision (PPV)**					
**Optimized**	**SVM**	**MLP**	**LR**	**XGB**	**ELM**
**MGO**	−0.028	+0.035	+0.036	+0.030	+0.036
**RUN**	−0.038	+0.026	+0.026	+0.020	+0.026
**CLPSO**	−0.018	+0.046	+0.047	+0.041	+0.047
**GA**	−0.043	+0.021	+0.021	+0.015	+0.021
**Accuracy**					
**Optimized**	**SVM**	**MLP**	**LR**	**XGB**	**ELM**
**MGO**	+0.012	+0.035	+0.037	+0.042 *	+0.038
**RUN**	+0.009	+0.033	+0.035	+0.039	+0.035
**CLPSO**	+0.016	+0.040	+0.042	+0.046 *	+0.043
**GA**	+0.004	+0.027	+0.029	+0.034	+0.030

**Table A4:** Statistical comparison on the SDB dataset between the four best optimized ELM models and key baseline classifiers. Each value reports the mean-metric difference between the optimized ELM and the corresponding baseline. Positive values favor the optimized ELM. Asterisks indicate Benjamini–Hochberg adjusted p<0.05 based on the Wilcoxon rank-sum test.

**ROC-AUC**					
**Optimized**	**SVM**	**MLP**	**LR**	**XGB**	**ELM**
**MEO**	+0.059 *	+0.001	+0.006	−0.036	+0.031
**HIWOA**	+0.047 *	−0.010	−0.005	−0.048 *	+0.019
**HGS**	+0.062 *	+0.005	+0.010	−0.032	+0.035
**HHO**	+0.042	−0.015	−0.010	−0.053 *	+0.015
**F1-Score**					
**Optimized**	**SVM**	**MLP**	**LR**	**XGB**	**ELM**
**MEO**	−0.026 *	−0.012 *	−0.025 *	−0.026 *	−0.010
**HIWOA**	−0.027 *	−0.014 *	−0.027 *	−0.027 *	−0.011 *
**HGS**	−0.028 *	−0.015 *	−0.028 *	−0.028 *	−0.013 *
**HHO**	−0.027 *	−0.014 *	−0.027 *	−0.027 *	−0.012
**Sensitivity (Recall)**					
**Optimized**	**SVM**	**MLP**	**LR**	**XGB**	**ELM**
**MEO**	−0.076 *	−0.026 *	−0.074 *	−0.076 *	−0.027 *
**HIWOA**	−0.074 *	−0.024 *	−0.073 *	−0.074 *	−0.026 *
**HGS**	−0.076 *	−0.026 *	−0.075 *	−0.076 *	−0.028 *
**HHO**	−0.073 *	−0.023 *	−0.072 *	−0.073 *	−0.024 *
**Precision (PPV)**					
**Optimized**	**SVM**	**MLP**	**LR**	**XGB**	**ELM**
**MEO**	+0.007	−0.003	+0.007	+0.007	+0.002
**HIWOA**	+0.003	−0.007	+0.003	+0.003	−0.002
**HGS**	+0.002	−0.007	+0.003	+0.002	−0.003
**HHO**	+0.002	−0.008	+0.002	+0.002	−0.003
**Accuracy**					
**Optimized**	**SVM**	**MLP**	**LR**	**XGB**	**ELM**
**MEO**	−0.031 *	−0.017 *	−0.030 *	−0.031 *	−0.013
**HIWOA**	−0.035 *	−0.021 *	−0.034 *	−0.035 *	−0.016
**HGS**	−0.037 *	−0.023 *	−0.036 *	−0.037 *	−0.018 *
**HHO**	−0.035 *	−0.021 *	−0.034 *	−0.035 *	−0.017
